# Valorization of Pitaya (*Selenicereus monacanthus*) Peels: Proof of Concept for Food Formulation

**DOI:** 10.1007/s11130-026-01507-y

**Published:** 2026-05-14

**Authors:** Karina Zanella Lodi, Ana Clara Fernandes Lima Rotini, Bárbara Richetti, Carina Cassini, Raquel Bridi, Catia Santos Branco

**Affiliations:** 1 Laboratory of Oxidative Stress and Antioxidants, Biotechnology Institute, University of Caxias do Sul, Caxias do Sul, Brazil; 2 Department of Life Sciences, University of Caxias do Sul, Caxias do Sul, Brazil; 3 Facultad de Ciencias Químicas y Farmacéuticas, Universidad de Chile, Santiago, Chile

**Keywords:** Cactaceae, Dragon fruit, Bioactive compounds, Sustainable food systems

## Abstract

Cacti by-products are rich in bioactive compounds, yet they remain largely underexplored in foods. This study investigated the nutritional and functional potential of red pitaya (*Selenicereus monacanthus)* peel flour in an innovative chocolate truffle composition. The phenolic composition, antioxidant activity, in vitro bioaccessibility, and intestinal permeability of flour were evaluated. It showed a significant content of bioactive flavonoids, with isorhamnetin, rutin, myricetin, quercetin, and luteolin as major identified compounds. In vitro passive permeability of polyphenol from flour was moderate (*P*_app_ = 5.24 ± 0.85 × 10^− 6^ cm/s). Chocolate truffles made with pitaya peel flour (BPF) showed an 86% increase in fiber, a 23% increase in ash content, a 27% reduction in lipids, and a 30% decrease in carbohydrates compared to those without addition. Adding peel flour increased the food’s antioxidant activity (3.5-fold) and phenolic composition (2.6-fold) compared to the control. Under simulated digestion, polyphenols’ bioaccessibility in BPF was higher in the ileum, and antioxidant activity was higher in the duodenal stage, compared to the gastric stage. Data indicates that pitaya peel flour is a promising input for developing foods.

**Supplementary Information:**

The online version contains supplementary material available at 10.1007/s11130-026-01507-y.

## Introduction

Latin American members of the Cactaceae family have received increased interest worldwide due to their significant potential for human nutrition, sustainable agriculture, and biotechnology. In this scenario, pitaya is a very promising fruit. Among the main species, red pitaya (*Selenicereus monacanthus* (Lem.) D.R. Hunt; syn. *Hylocereus lemairei* (Hook.) Britton & Rose) is one of the most studied. The fruit is valued for providing essential micronutrients, antioxidants, and bioactive compounds, including flavonoids [1]. Pitaya peels account for approximately one-third of the fruit’s weight and are typically discarded. However, previous studies have demonstrated that peel is a good source of polyphenols, compounds with substantial antioxidant activity [2, 3]. In the context of climate change and food insecurity, the use of underutilized plants and their non-edible parts plays a crucial role in modern nutrition and biotechnological agendas. To minimize seasonal fruit waste, dehydration techniques are useful to preserve their antioxidant and nutritional properties. Hence, producing flour is a valuable strategy for food supplementation, as incorporating powdered fruits and byproducts into foods can enhance the health-promoting properties of the resulting formulations [4]. Fruit waste (*e.g*., peels) is among the most valued raw materials, with applications in a wide range of products, including cookies and bakery products. Until now, the addition of by-products in candy and sweets has been limited. Chocolate truffles are extremely popular among people of all ages, particularly younger audiences. They have no nutritional value because they are high in sugar and fat, so strategies to modify ingredient composition represent a valuable and innovative alternative. The present study aimed to produce pitaya peel flour and incorporate it into a chocolate truffle, to assess its composition and phenolic bioaccessibility under simulated digestion. We hypothesize that pitaya peel flour can enhance the nutritional profile and maintain antioxidant stability within a lipid-rich confectionery matrix, compared to a control formulation. This work provides proof of concept for the valorization of fruit processing byproducts as functional ingredients, contributing to more sustainable and circular food strategies.

## Materials and Methods

This section is presented in the Supplementary Materials.

## Results and Discussion

The growing demand for sustainable diets has increased interest in food developed from agro-industrial byproducts. In this context, the present study investigates the use of red pitaya peel to produce flour for food incorporation, emphasizing the valorization of Cacti derivatives. Moisture content is a crucial indicator of flour quality, directly affecting food preservation and stability processes. Pitaya flour exhibited values within the expected range for vegetable sources (< 15 g/100 g) and was maintained over a 150-day storage period (Table S1). Pitaya peel flour also displayed significant antioxidant activity (AA), which was maintained for up to 150 days of storage, while total phenolic content (TPC) remained stable for 60 days (Table S2). Correlation analyses showed a negative correlation between TPC and moisture (*r* = -0.483, *p* = 0.003), indicating that higher moisture levels were associated with lower phenolic content. A low moisture content is essential for maximizing the preservation of bioactive compounds in flour, thereby ensuring a higher-quality, more stable product. UPLC-MS/MS demonstrated isorhamnetin, the 3-*O*-methylated metabolite of quercetin, was the major compound (166.48 ± 21.42 mg/100 g), followed by myricetin (9.227 ± 3.158 mg/100 g), rutin (1.848 ± 0.315 mg/100 g), quercetin (1.037 ± 0.362 mg/100 g), and luteolin (0.348 ± 0.127 mg/100 g) in pitaya flour. Previously, a study identified rutin, isorhamnetin, and quercetin in the aqueous extract of pitaya (*Stenocereus thurberi*) pulp [5]. Furthermore, myricetin and quercetin have already been detected in *Hylocereus* species [6]. The dominance of isorhamnetin in flour composition has functional advantages, as methylation reduces the number of free hydroxyl groups available for redox reactions, thereby improving matrix stability and reducing susceptibility to degradation [7]. Moreover, methoxylated flavonoids are described as more stable and often more lipophilic, which influences their behavior in food systems and during digestion [7, 8]. Polyphenols in pitaya flour exhibited a lower apparent permeability (P_app_) than the positive control (5.24 ± 0.85 × 10^− 6^ cm/s; *p* < 0.01) but were 80% higher than the negative control (*p* < 0.01), indicating intermediate permeability (Figure S2). PAMPA is a useful assay for estimating polyphenol permeability and predicting passive absorption from complex matrices [9–11]. It is considered one of the most effective and versatile screening tools for early drug discovery. Because the membrane used in the assay is artificial, only passive transport mechanisms occur, unlike in cell-based assays [11]. The permeability classification can be defined as low (< 5 × 10^− 6^ cm/s), moderate (5–10 × 10^− 6^ cm/s), and good (> 10 × 10^− 6^ cm/s) [12]. Our findings indicate that pitaya peel flour is a source of polyphenols with moderate passive permeability, suggesting a potential for passive diffusion across lipid-like membranes and supporting its application as a functional ingredient in food systems. To achieve this, *brigadeiro*, a traditional Brazilian chocolate truffle (BPF), was used (Figure S1). Data about this composition is shown in Figure S3. An increase of about 30% in mineral content (ashes) was found in BPF. Regarding lipids, a reduction of approximately 1.5 times in BPF was observed. Fibers derived from fruits offer technological advantages over those from cereals, which are commonly used to enrich foods. The addition of pitaya peel significantly increased the fiber content compared to the control. Similarly, a significant increase in fiber content was reported in cookies made with pitaya peel [13]. Incorporating fiber in foods is useful for fat retention capacity and reducing caloric value. In fact, reductions of 30 and 37% in carbohydrate and energy values were found in BPF. These results are significant, as reducing sugar content may benefit young people, who frequently consume foods that provide empty calories, leading to health issues such as obesity, diabetes, and poor dental health. Interactions between polyphenols and truffle matrix may influence both their stability and gastrointestinal behavior. Phenolic compounds can form complexes with fiber, carbohydrates, lipids, and proteins, thereby significantly affecting their bioaccessibility [14, 15]. The lipid-rich nature of cocoa truffles may favor the localization of certain phenolic compounds at lipid-water interfaces, facilitating their gradual release and improving bioaccessibility. In fact, it has been shown that the presence of lipids in the food matrix can increase micelle formation during intestinal digestion, favoring the incorporation of nonpolar polyphenols and, consequently, increasing their soluble (bioaccessible) fraction in the digested material [16]. These combined interactions suggest that a complex food matrix may contribute to improved retention and a more gradual release of polyphenols during digestion, as observed in the present study. The addition of pitaya flour in chocolate truffles resulted in a 2.6-fold increase in TPC and a 3.5-fold increase in AA (Table S3). Similar results were reported by Ramashia et al. [17], who observed increases in TPC and AA in biscuits enriched with *Parinari curatellifolia* peel. To measure the polyphenol bioaccessibility and AA from pitaya-enriched truffles, simulated gastrointestinal digestion was carried out (Table S4). It was observed that in the last stage of digestion (ileum), the sample showed values similar to those of the oral phase. In the jejunal and duodenal phases, an increase in AA was observed. The study by Plaitho et al. [18] reported the antioxidant activity of mango peel extract and its bioaccessible fraction in muffins, thus corroborating our findings. Additionally, methoxylated flavonoids, such as isorhamnetin, the major phenolic compound in pitaya flour, are reported to be favored during digestion, which may facilitate their absorption and enhance oral bioaccessibility [7, 8]. Despite these promising findings, however, this study has some limitations. The in vitro digestion and PAMPA models employed in this study provide simplified estimates of bioaccessibility and passive diffusion. These approaches do not account for active transport mechanisms, intestinal metabolism, or the complexity of the epithelial barrier. Therefore, the results should be interpreted as preliminary indicators, and further studies using cell-based systems or in vivo models are required to confirm biological relevance.

## Conclusions

Pitaya peel flour, when incorporated into chocolate truffles, increased total phenolic content and antioxidant capacity. Improvements in the product’s nutritional composition and phenolic bioaccessibility were also observed, supporting its potential use in a confectionery food matrix. The flour was characterized as a source of phenolic compounds, including isorhamnetin, rutin, myricetin, quercetin, and luteolin. Converting pitaya peel into flour represents a viable strategy for valorizing agro-industrial byproducts, reducing waste, and providing a stable ingredient that is independent of fruit seasonality.

## Supplementary Information

Below is the link to the electronic supplementary material.

Supplementary Material 1 (DOCX 3.71 MB)

## Data Availability

No datasets were generated or analysed during the current study.
