# Supplementary material for: Valorization of Pitaya (Selenicereus monacanthus) Peels: Proof of Concept for Food Formulation
Source: Plant Foods Hum Nutr. 2026 May 14;81(2):58. doi: 10.1007/s11130-026-01507-y (PMC13171682; doi:10.1007/s11130-026-01507-y)
Supplement: Supplementary file 1 — Supplementary Material 1 (DOCX 3.71 MB) [file 11130_2026_1507_MOESM1_ESM.docx]

*Supplemental material*

***Valorization of pitaya (Selenicereus monacanthus) peels: proof of concept for food formulation***

Karina Zanella Lodi^a^, Ana Clara Fernandes Lima Rotini^b^, Bárbara Richetti^b^, Carina Cassini^a^, Raquel Bridi^c^ and Catia Santos Branco^ab^*

*^a^ Laboratory of Oxidative Stress and Antioxidants, Biotechnology Institute, University of Caxias do Sul, Caxias do Sul, Brazil; ^b^Department of Life Sciences, University of Caxias do Sul, Caxias do Sul, Brazil; ^c^ Facultad de Ciencias Químicas y Farmacéuticas, Universidad de Chile, Santiago, Chile*

*Corresponding Author: Laboratory of Oxidative Stress and Antioxidants. Biotechnology Institute. University of Caxias do Sul, Brazil. Central Campus Phone: +55 54 32182105. E-mail: [csbranc1@ucs.br](mailto:csbranc1@ucs.br) ORCID:0000-0003-3709-3004.

**Abstract**

Cacti by-products are rich in bioactive compounds, yet they remain largely underexplored in foods. This study investigated the nutritional and functional potential of red pitaya (*Selenicereus monacanthus)* peel flour in an innovative chocolate truffle composition. The phenolic composition, antioxidant activity, *in vitro* bioaccessibility, and intestinal permeability of flour were evaluated. It showed a significant content of bioactive flavonoids, with isorhamnetin, rutin, myricetin, quercetin, and luteolin as major identified compounds. *In vitro* passive permeability of polyphenol from flour was moderate (*P*_app_ = 5.24 ± 0.85 × 10^-6^ cm/s). Chocolate truffles made with pitaya peel flour (BPF) showed an 86% increase in fiber, a 23% increase in ash content, a 27% reduction in lipids, and a 30% decrease in carbohydrates compared to those without addition. Adding peel flour increased the food's antioxidant activity (3.5-fold) and phenolic composition (2.6-fold) compared to the control. Under simulated digestion, polyphenols’ bioaccessibility in BPF was higher in the ileum, and antioxidant activity was higher in the duodenal stage, compared to the gastric stage. Data indicates that pitaya peel flour is a promising input for developing foods.

**Keywords:** Cactaceae; dragon fruit; bioactive compounds; sustainable food systems

**3. Experimental**

**3.1 Red pitaya**

Pitayas (*Selenicereus monacanthus* (Lem.) D.R. Hunt; syn. *Hylocereus lemairei* (Hook.) Britton & Rose) were collected in May 2020 (27◦ 35’ 23.172’’ S, 48◦ 43’ 2.568’’ W). A plant exsiccate was deposited in the Herbarium of the University of Caxias do Sul (HUCS 50878). After visual selection, the fruits were classified and cleaned in a 1% chlorinated solution for 15 minutes. They were washed with running water and dried on filter paper for subsequent weighing and pulping.

**3.2 Preparation of pitaya peel flour and nutritional composition evaluation**

The peels were dried in a forced circulation oven at 45 ± 5 °C and then ground in a knife mill (flour). The sample was then stored in a desiccator at room temperature, with protection from light and oxygen, for the entire analysis period. The flour was kept in a drying oven (105 °C) for 3 hours to determine the moisture content. It was monitored from 0 to 150 days. To quantify ashes, it was incinerated (500 ºC) for 4 hours. Protein (Kjeldahl method) and dietary fiber were also quantified according to AOAC [1]. Fats were estimated by the Bligh and Dyer method [2]. Carbohydrates were obtained by difference, and energy value was calculated using conversion (Carbohydrates – 4 kcal/g; Proteins – 4 kcal/g; Lipids – 9 kcal/g).

**3.3 Preparation of pitaya peel extract**

Phenolics were extracted from flour peels using water as solvent (1%; w/v), in a condenser under a reflux system, as previously described [3]. The extract was lyophilized and stored in a desiccator protected from light exposure until analysis.

**3.3.1 Antioxidant activity and phenolic composition**

Antioxidant activity and total phenolic content were evaluated in the lyophilized extract over a 150-day storage period. Antioxidant activity was performed through the free radical scanning capability of 2,2-75 diphenyl,1-picrylhydrazyl (DPPH^•^) [4]. Briefly, 100 µL of the extract was added to 400 µL of Tris/HCl buffer (pH 7.0) and 500 µL of DPPH^•^ ethanol solution (500 µM) under dark conditions for 30 min. Absorbance was measured at 517 nm, and results were expressed in % DPPH^•^ inhibition. To evaluate total phenolic content, the Folin-Ciocalteu method described by Singleton and Rossi [5] was used. Folin reagent (1N) was diluted in distilled water at a concentration of 1:1, in which 100 µL of extract was added to 500 µL of Folin-Ciocalteu solution (1N) and 400 µL of sodium carbonate (Na_2_CO_3_) (7.5% w/v). After 30 min, the absorbance was measured spectrophotometrically at 765 nm, and the results were expressed as mg/100 g dry weight.

**3.3.2 UPLC-MS/MS Analysis**

Phenolic compounds were identified in the extract using an ABSciex triple Quad 4500 mass spectrometer equipped with an electrospray (TurboV) interface coupled to an Eksigent Ekspert Ultra LC100 with an Ekspert Ultra LC100-XL autosampler system (AB/Sciex Concord, ON, Canada). Chromatographic separation was carried out by employing a gradient elution with (A) 0.1% formic acid and (B) methanol as the mobile phase, using the following protocol: 0–1 min, 5% B; 1–12 min, 5–50% B; 12–13 min 50–50% B; 13–14 min, 50–5% B; and 14–15 min, 5% B. The instrument was operated using an injection volume of 10 µL, a flow rate of 0.5 mL/min, and an end-capped column (LiChrospher 100 RP-18; 125 mm x 4 mm i.d., 5 µm; Merck, Darmstadt, Germany) maintained at 50 °C. Calibration curves for quantification were constructed using commercially available standards [6]. Results were expressed as mg/100 g dry weight.

**3.3.3 Parallel Artificial Membrane Permeability Assay (PAMPA)**

PAMPA was performed according to Sierpe et al. [7], using a transwell plate with one donor (lower) and another acceptor (upper), separated by a semipermeable polyvinylidene fluoride membrane. Twenty mg/mL phosphatidylcholine in dodecane solution was deposited on the acceptor plate and left for 5 min. Subsequently, 300 μL of phosphate-buffered saline (PBS) at pH 7.4 was added. In the donor well, 300 μL of extract (prepared as described in section 3.3) was deposited. Solutions of 10mg/mL thiopental and Evans blue in PBS were used as positive and negative controls, respectively. The plate was sealed with parafilm and shaken at 300 rpm for 24 h at 37 °C. Effective permeability (Pe) was expressed in 10^-6^ cm/s based on total phenolic quantification measured by the Folin-Ciocalteu method, as described in section 3.3.1.

**3.4 Preparation of chocolate truffles**

Chocolate truffles (*brigadeiros*) were produced at the Dietary Techniques Laboratory at the University of Caxias do Sul (Brazil). Two formulations were produced: one containing 40 g of peel flour and the other without peel flour (control). To make it, condensed milk (395 g), cocoa powder (70%; 60 g), and butter (20 g) were mixed in a stainless-steel pan. Then, it was placed over medium heat to obtain the common point (detaching from the bottom). Afterwards, it was poured into a glass container to cool before making balls. The amount of pitaya flour was defined based on previous tests.

**3.4.1 Nutritional composition of chocolate truffles**

The quantification of moisture, ash, crude fiber, total lipids, protein, carbohydrate, and energy value was carried out according to methodologies previously described in section 3.2.

**3.4.2 Extract preparation for measuring total phenolic content and antioxidant activity**

To extract polyphenols from chocolate truffles and assess their antioxidant activity, an extractive solution composed of ethanol, acetic acid, and water in a 50:8:42 ratio was used, according to Šaponjac et al. [8], with modifications. The truffles were homogenized with the extractive solution (100 mg/mL) in an orbital shaker (TE 420, Tecnal, Brazil) for 15 minutes, then centrifuged at 6000 rpm for 10 minutes. The supernatant was collected and filtered through 0.45 µm membranes.

**3.4.3 Total phenolic content (TPC) and antioxidant activity**

The quantification of total phenolic content and antioxidant activity of chocolate truffles was carried out according to methodologies previously described in section 3.3.1.

**3.4.4 *In vitro* gastrointestinal digestion**

The TPC bioaccessibility assay, simulating physiological conditions in the upper digestive tract (mouth, stomach, and small intestine), was performed with modifications [9]. Supernatants (bioaccessible fractions) were used for TPC analysis (Folin Ciocalteu) and antioxidant activity (DPPH), as previously described. In both assays, the absorbance of the control (without pitaya flour) was measured. The bioaccessibility index (BI) was calculated as the percentage of polyphenols/antioxidant activity remaining in the bioaccessible fraction related to the original non-digested sample, and the results are expressed as % (Eq. 1).

*BI* = $\frac{Cb}{Ca}$ × 100

Cb is the concentration of the TPC or its antioxidant activity at the end of a digestion step, and Ca is the concentration of the TPC or its antioxidant activity in the sample before the digestion process.

**3.5 Statistical analysis**

Analyzes were performed in triplicate, and results were expressed as mean ± standard deviation. Data were analyzed using variance (One-way ANOVA) and Tukey's post hoc test or Student's t-test, with a probability level of less than 5% (*p* < 0.05) in SPSS version 23.

**Results**

**
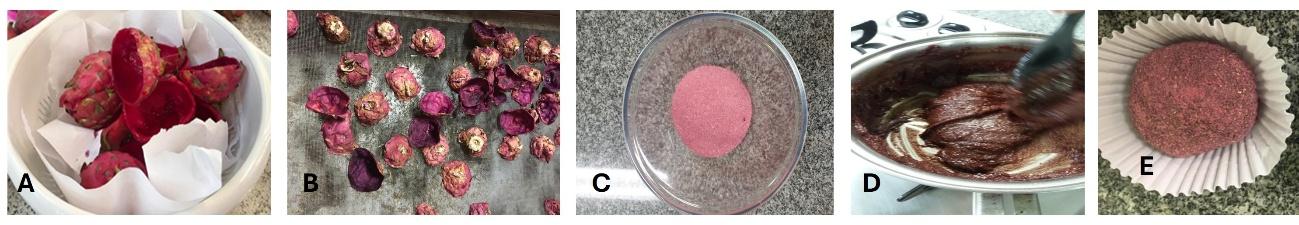
**

**Figure S1.** Stages of preparing the typical Brazilian truffles, called *brigadeiros*. Pitaya peels *in natura* (A) and dried (B); flour (C); common *brigadeiro* point (D); and ready-made *brigadeiro* (E).

**Table S1.** Moisture monitoring in pitaya peel flour over a 150-day storage period.

|  |  | **Moisture (g/100g)** |
| --- | --- | --- |
| **Storage time (days)** | **0** | 5.47 ± 0.41^a^ |
|  | **30** | 7.88 ± 0.39 ^ab^ |
|  | **60** | 8.99 ± 1.58 ^bc^ |
|  | **90** | 11.13 ± 2.83 ^cd^ |
|  | **120** | 12.00 ± 0.20 ^d^ |
|  | **150** | 12.37 ± 0.03 ^d^ |

Legend: Values are expressed as mean ± standard deviation. Different letters indicate statistical differences by Analysis of Variance (ANOVA) and Tukey's post-hoc test between storage times (*p*<0.05).

**Table S2.** Antioxidant activity (AA) and total phenolic content (TPC) in pitaya peel flour over a 150-day storage period.

| **AA (% DPPH inhibition)** | | |
| --- | --- | --- |
| **Storage time (days)** | **0** | 62.47 ± 0.50 ^ab^ |
|  | **30** | 65.25 ± 2.17 ^a^ |
|  | **60** | 61.66 ± 4.60 ^ab^ |
|  | **90** | 61.50 ± 1.30 ^ab^ |
|  | **120** | 58.60 ± 1.39 ^b^ |
|  | **150** | 57.49 ± 1.31 ^b^ |
| **TPC (mg/100g)** | | |
| **Storage time (days)** | **0** | 388.03 ± 11.20 ^a^ |
|  | **30** | 367.58 ± 11.78 ^a^ |
|  | **60** | 335.08 ± 4.71 ^c^ |
|  | **90** | 340.08 ± 3.54 ^bc^ |
|  | **120** | 346.47 ± 5.89 ^b^ |
|  | **150** | 331.42 ± 11.98 ^c^ |

Legend: Values are expressed as mean ± standard deviation. Different letters indicate statistical differences by Analysis of Variance (ANOVA) and Tukey's post-hoc test between storage times (*p* < 0.05).


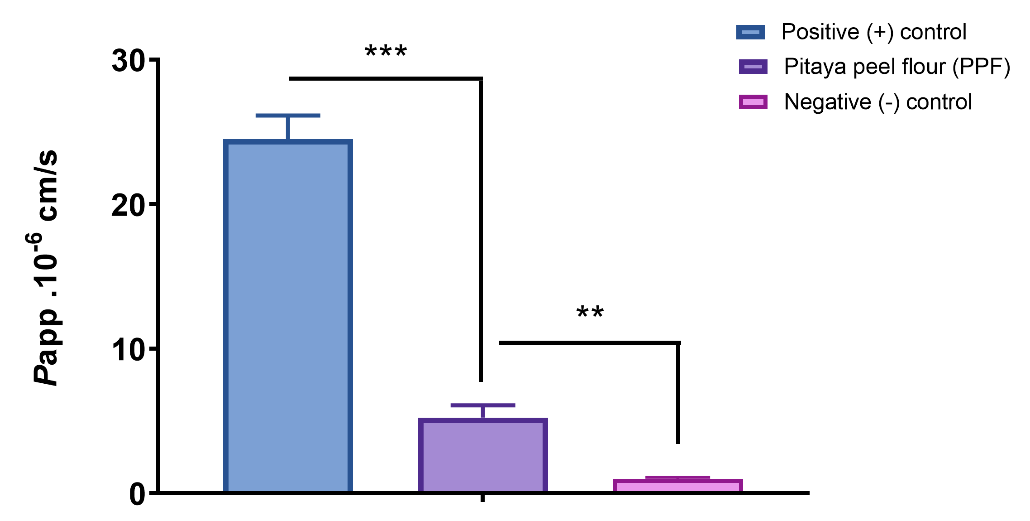


**Figure S2.** Parallel Artificial Membrane Permeability Assay (PAMPA) in pitaya flour. Legend: Asterisks (*) indicate statistical differences by Analysis of variance (ANOVA) Tukey's post-hoc test between samples (***p* < 0.05; ***p* < 0.01).

**
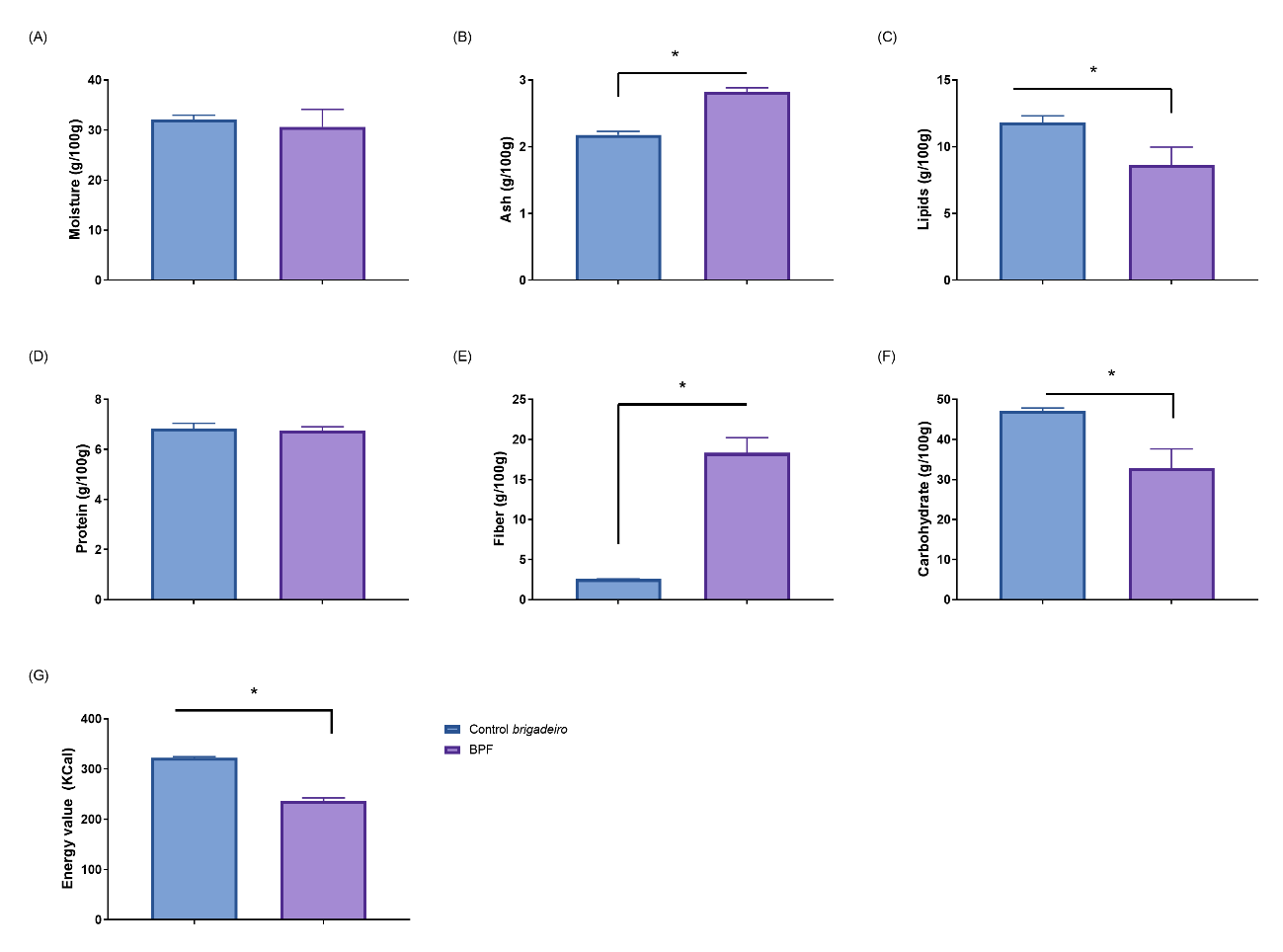
**

**Figure S3.** Assessment of moisture content (A), ash (B), lipids (C), proteins (D), fibers (E), carbohydrates (F), and energy value (G) in chocolate truffle (*brigadeiro*) containing pitaya peel (BPF) compared to control (without pitaya peel flour). Legend: Values are expressed as mean ± standard deviation. Asterisks (*) indicate statistical differences by *t*-test between samples (*p* < 0.05).

**Table S3.** Total phenolic content (TPC) and antioxidant activity (AA) expressed as percentage of DPPH scavenger (%) of chocolate truffle (*brigadeiro)* without and with pitaya peel flour (BPF).

|  | **TPC (mg/100g)** | **AA (%)** |
| --- | --- | --- |
| Control | 122 ± 3.00 | 11.37 ± 0.06 |
| BPF | 315 ± 8.00* | 40.04 ± 0.28* |

Legend: AA (Antioxidant Activity); TPC (Total Phenolic Content). Values ​​are expressed as mean ± standard deviation. Asterisks (*) indicate statistical differences by *t*-test between samples (*p* < 0.05).

**Table S4.** TPC bioaccessibility (%) and antioxidant activity retention (%) of chocolate truffle (*brigadeiro*) containing pitaya peel flour (BPF) after *in vitro* digestion.

|  | **TPC (%)** | **AA (%)** |
| --- | --- | --- |
| **Oral** | 73.34± 12.92^a^ | 21.17 ± 7.72^d^ |
| **Gastric** | 40.79 ± 0.94^c^ | 29.53 ± 1.02^cd^ |
| **Duodenal** | 39.54 ± 2.96^c^ | 42.01 ± 4.56^ab^ |
| **Jejunal** | 51.76 ± 8.67^b^ | 37.93 ± 2.02^bc^ |
| **Ileal** | 77.39 ±1.88^a^ | 36.16 ± 4.22^ab^ |

Legend: AA (Antioxidant Activity); TPC (Total Phenolic Content). Values ​​are expressed as mean ± standard deviation. Different letters indicate statistical differences by Analysis of Variance (ANOVA) and Tukey's post-hoc test between digestion stages (*p* < 0.05).

**Supplementary References**

1. AOAC (2023) Official Methods of Analysis: 22nd Edition (2023). Off Methods Anal AOAC Int 22nd Ed 0

2. Bligh, E.G. and Dyer WJ (1959) Canadian Journal of Biochemistry and Physiology. Can J Biochem Physiol 37:

3. Lodi K, Cappelari M, Pilatti GC, et al (2022) Pre-clinical evidence for the therapeutic effect of Pitaya (Hylocereus lemairei) on diabetic intestinal microenvironment. Nat Prod Res 0:1–7. https://doi.org/10.1080/14786419.2022.2110091

4. Yamaguchi T, Takamura H, Matoba T, Terao J (1998) Hplc method for evaluation of the free radical-scavenging activity of foods by using 1,1-diphenyl-2-picrylhydrazyl. Biosci Biotechnol Biochem 62:1201–1204. https://doi.org/10.1271/bbb.62.1201

5. Singleton VL, Rossi JAJ (1965) Colorimetry to total phenolics with phosphomolybdic acid reagents. Am J Enol Vitic 16:144–58

6. de Camargo AC, Alvarez AC, Arias-Santé MF, et al (2022) Soluble Free, Esterified and Insoluble-Bound Phenolic Antioxidants from Chickpeas Prevent Cytotoxicity in Human Hepatoma HuH-7 Cells Induced by Peroxyl Radicals. Antioxidants 11:. https://doi.org/10.3390/antiox11061139

7. Sierpe R, Noyong M, Simon U, et al (2017) Construction of 6-thioguanine and 6-mercaptopurine carriers based on βcyclodextrins and gold nanoparticles. Carbohydr Polym 177:22–31. https://doi.org/10.1016/j.carbpol.2017.08.102

8. Tumbas Šaponjac V, Ćetković G, Čanadanović-Brunet J, et al (2016) Sour cherry pomace extract encapsulated in whey and soy proteins: Incorporation in cookies. Food Chem 207:27–33. https://doi.org/10.1016/j.foodchem.2016.03.082

9. Bridi R, Echeverría J, Larena A, et al (2022) Honeybee Pollen From Southern Chile: Phenolic Profile, Antioxidant Capacity, Bioaccessibility, and Inhibition of DNA Damage. Front Pharmacol 13:1–12. https://doi.org/10.3389/fphar.2022.775219
